# Malignant Transformation in Ollier's Disease: A Novel Stem for a Tibial Megaprosthesis

**DOI:** 10.7759/cureus.34057

**Published:** 2023-01-22

**Authors:** Vincent Y Ng, Philip Khoury

**Affiliations:** 1 Department of Orthopedics, University of Maryland Medical Center, Baltimore, USA; 2 Department of Orthopedics, School of Medicine, University of Maryland, Baltimore, USA

**Keywords:** endoprosthesis, megaprosthesis, tibial reconstruction, chondrosarcoma, malignant transformation, enchondroma, ollier’s disease

## Abstract

Ollier’s disease is a rare syndrome characterized by multiple enchondromas with the potential for malignant transformation. The treatment for secondary chondrosarcoma is surgical resection, which can be a morbid procedure depending on the location and size of the tumor. We present a successful limb salvage in which the majority of the tibia was removed and replaced with a megaprosthesis. The complex reconstruction in this case required the use of a novel uncemented stem.

## Introduction

Ollier’s disease is a form of multiple enchondromatosis. It is a rare disorder caused by de novo somatic mutations and is characterized by numerous enchondromas predominantly in the metaphysis and diaphysis of bones, particularly in the hands, feet, and long bones of the extremities [1]. Due to the varying severity and asymmetrical nature of the enchondromas, Ollier’s disease can cause limb deformities due to impaired growth or bone curvature. Patients with Ollier’s disease have a 25% risk of malignant transformation by the fourth decade of life and up to a 40% lifetime risk [2,3]. 

Reconstruction after surgical resection of malignant bone tumors is often complex and may require innovative techniques depending on the anatomic location. Segmental defects in the bone can be replaced using autograft, allograft, off-the-shelf megaprosthesis, custom-made megaprosthesis, or an alloprosthetic composite (APC). The stability of bone grafts requires internal fixation and eventual healing of the bony junction. Megaprostheses are affixed to native bone with either cemented or uncemented stems. Decision-making must be individually tailored to the clinical situation and the particular risks and benefits of each reconstructive method. This case report describes a novel solution using a unique stem design for reconstructing a near-total tibial defect, a challenging situation for which there is limited existing literature.

## Case presentation

**Figure 1:**
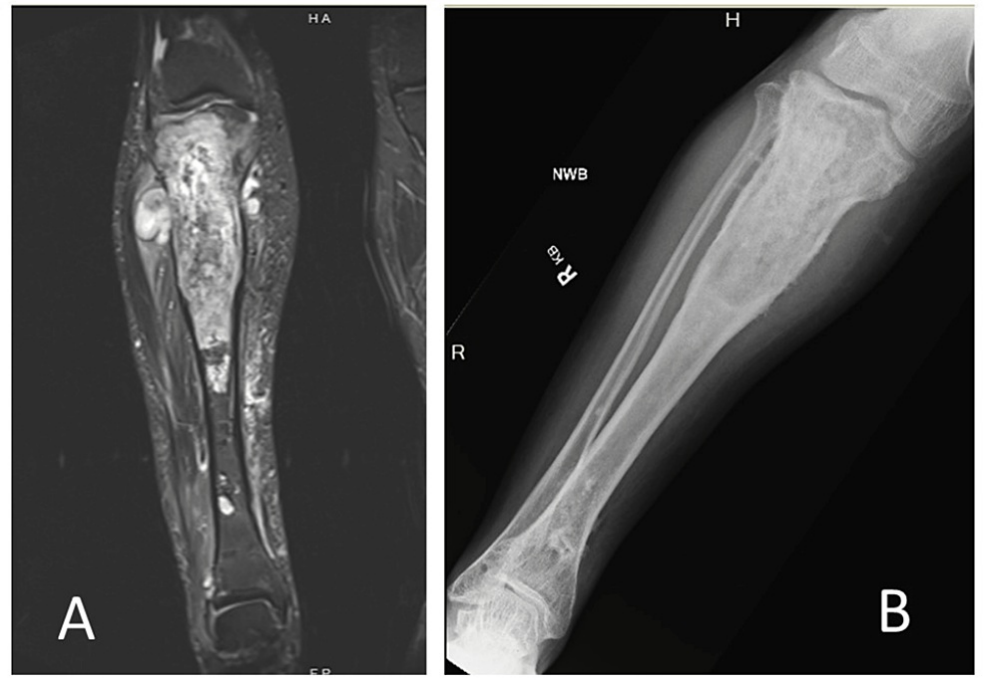
MRI (A) and radiograph (B) of the tibia. MRI: magnetic resonance imaging.

A 44-year-old male with a known history of Ollier’s disease presented with progressively increasing pain over two years. He reported swelling in his right proximal tibia and worsening discomfort during ambulation. On physical exam, he had a palpable enlargement of his proximal tibia and multiple healed scars over the distal tibia from prior external fixation pins. Further evaluation with imaging was indicated. Magnetic resonance imaging (MRI) and radiographs demonstrated an aggressive tumor with multiple areas of extraosseous extension (Figure 1A and B).

**Figure 2:**
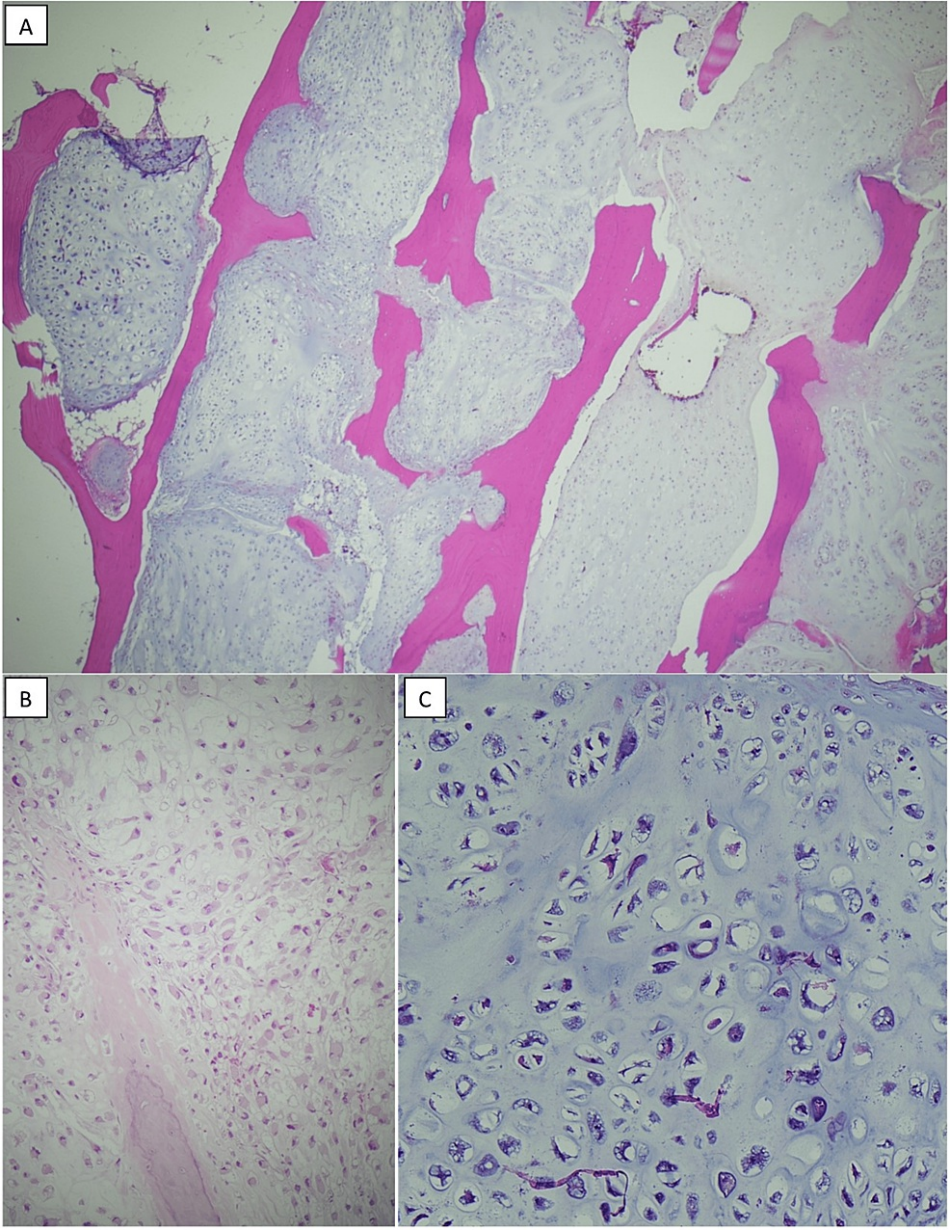
Histologic images from high-grade chondrosarcoma, H&E stains. (A) Low-power view demonstrates tumor percolation or perfusion through bone, a growth pattern indicative of malignancy in chondrosarcoma (20x magnification). (B, C) Areas of the tumor demonstrate chondrocyte hypercellularity and cytologic atypia, to a degree diagnostic of high histologic grade (B: 200x magnification, C: 400x magnification).

Histology from a core needle biopsy was consistent with high-grade chondrosarcoma (Figure 2).

 A computed tomography (CT) scan of his chest demonstrated no evidence of lung metastases and a whole-body bone scan demonstrated no other areas of significant activity aside from several foci more distal in the ipsilateral tibia. He had a pre-existing limb-length discrepancy due to underdevelopment of the right lower extremity and had limited motion in his ipsilateral ankle.

 Despite the challenges and risks presented by this tumor and its anatomic location, the patient declined amputation and wished to proceed with limb preservation. There were several foci of cartilage tumor visible, extending more distally in the tibia. Preservation of the distal third of the tibia would provide more bone stock for reconstruction; however, it was deemed to be a riskier option. A needle biopsy of these lesions was felt not to be effective in this situation because of the heterogeneity of chondroid lesions and the histological challenges of differentiating chondrosarcoma from enchondroma with a needle biopsy. A separate curettage of the lesions distal to the primary tumor for histological analysis prior to resection of the primary tumor was considered but not performed for a couple of reasons. First, there was not a wide area of normal bone between the distal lesions and the primary tumor to delimit the area of curettage. Second, the creation of a long bone window to facilitate curettage would have compromised future stem fixation. Thus, these distal lesions were included in the resection plan on account of their proximity to the primary tumor and their potential for being skip metastases. Only approximately 5 cm of distal tibial bone would remain after resection.

**Figure 3:**
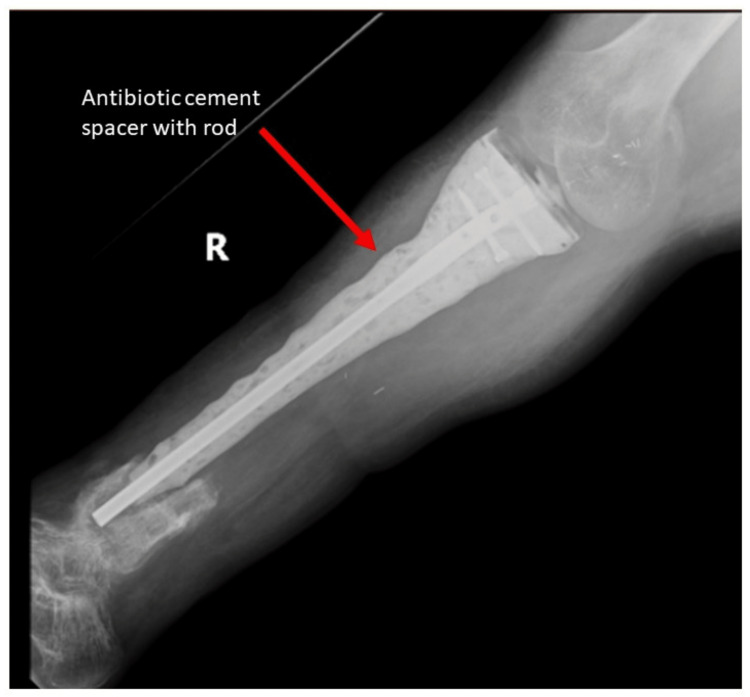
Radiograph after resection of tumor and placement of spacer.

 His surgeries were planned as staged procedures to reconstruct the tibia and knee with a megaprosthesis and to perform a tibio-talo-calcaneal (TTC) arthrodesis using the stem. In the first stage, the tumor was resected, and the defect was temporarily reconstructed with an antibiotic-laden cement spacer (Figure 3).

**Figure 4:**
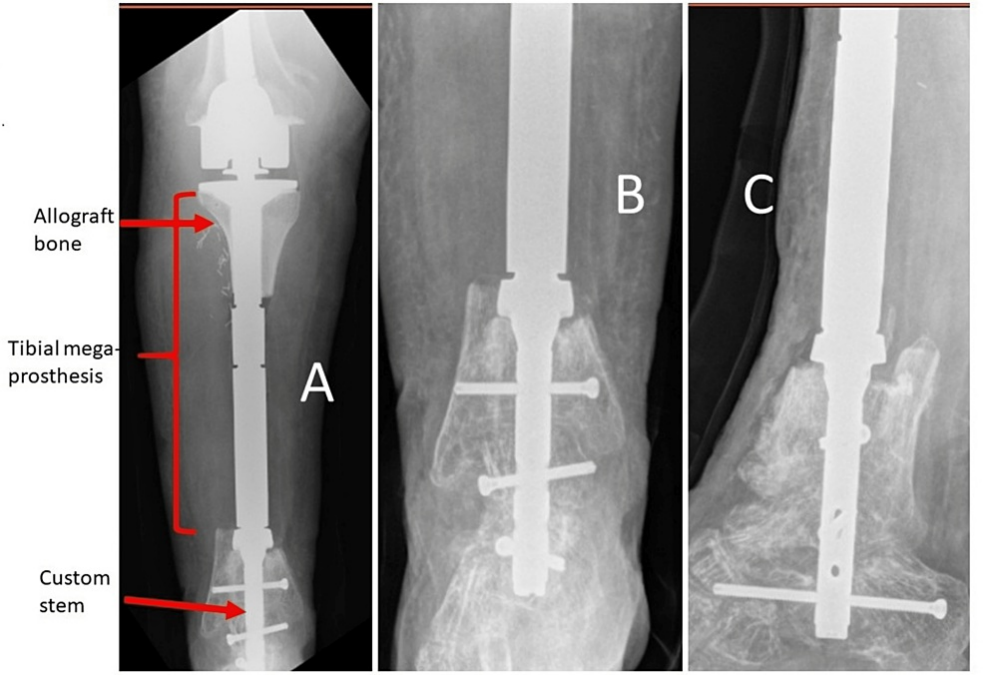
Radiographs of prosthetic (A) and the stem (B, C) for the tibial megaprosthesis.

 This allowed his soft tissue envelope to heal and recover. Because of the resection of the anterior tibial artery with the tumor and the underdevelopment of the medial gastrocnemius and posterior vasculature in his right leg, rotation or free-flap coverage was not felt to be a safe option by both a vascular surgeon and a microsurgeon. Postoperative scans were provided to Zimmer-Biomet (Warsaw, IN) for their design and custom-stem production. Because of the short segment of the tibia and the ankle and subtalar joints, a traditional uncemented press-fit or cemented stem was not felt to be feasible. A Compress® (Zimmer-Biomet, Warsaw, IN) was considered, particularly with the short anchor plug, but the length of remaining bone available in the distal tibia would be very close to the minimally feasible limit for this device. Implant failure due to fracture has been described previously and was also a concern in this situation [4]. Once the custom stem was made, the patient underwent surgery. For the knee joint, a rotating-hinge total knee arthroplasty was used. To recreate the patient’s missing patellar tendon and extensor mechanism, an allograft proximal tibia with a full extensor mechanism was used as a composite with the prosthesis. The custom stem to hold the tibial megaprosthesis in place was designed by the company as a cylindrical uncemented stem with proximal ingrowth, a collar, and interlocking holes similar to a traditional TTC fusion nail (Figure 4).

**Figure 5:**
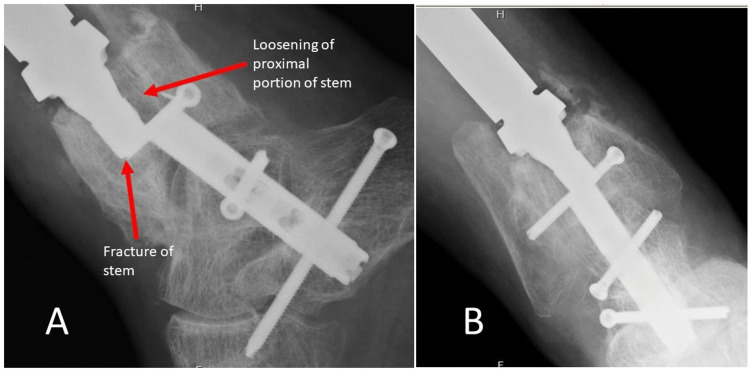
Radiographs of the failed stem (A, B).

 The patient did well initially, but 10 months after reconstruction, radiographs demonstrated a fracture of the custom stem through the proximal interlocking hole and loosening of the proximal portion of the stem (Figure 5).

**Figure 6:**
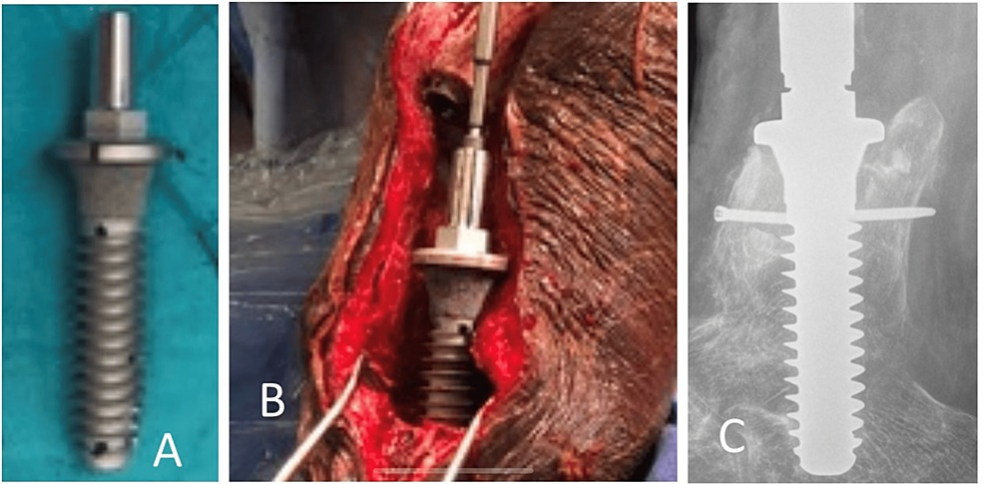
Intraoperative photographs (A, B) and postoperative radiograph (C) of the custom stem based on a senior author's design.

 The patient was unable to ambulate. Subsequently, the senior author provided a novel prosthetic stem design to the manufacturer. It included a collar, threads, and interlocking holes and was successfully implanted after the fabrication of the implant (Figure 6).

 The patient required a reoperation one month later due to a complication with the total knee articulation. However, this was unrelated to the stem, and the stem did not require any revision or adjustment.

**Figure 7:**
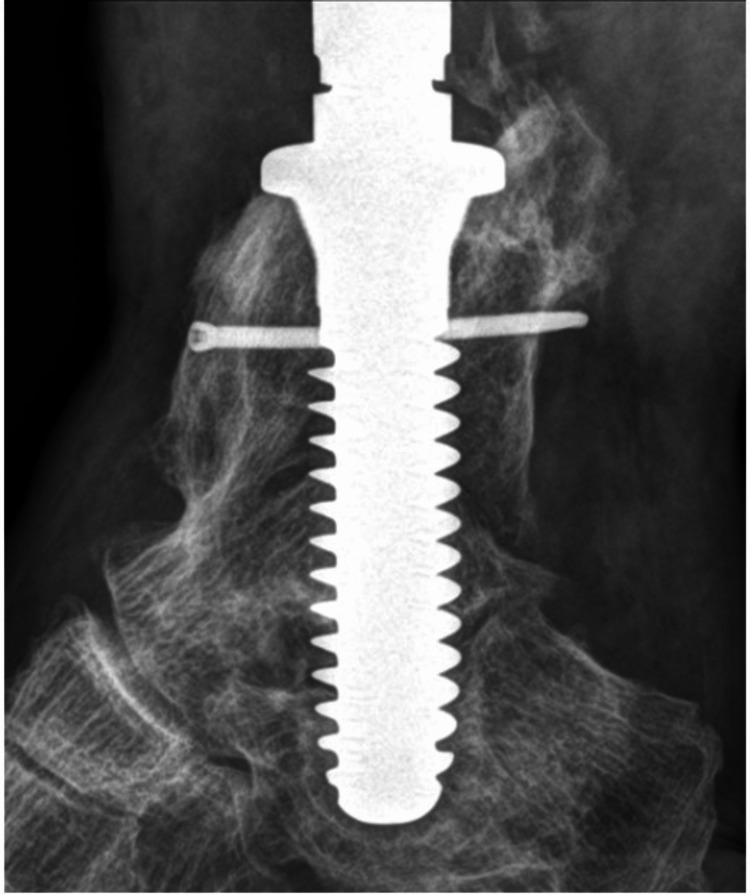
Radiograph demonstrating the stability of the stem at 2.5 years after implantation.

 At the latest follow-up, 2.5 years after the final reconstruction, the patient is ambulating, and radiographs demonstrate no evidence of loosening (Figure 7).

## Discussion

Reconstruction of major bone resections in the tibia has higher complication and failure rates for both allograft and megaprosthetic options than in other areas of the skeleton [5,6]. Limited remaining bone after resection is a risk factor for inadequate fixation and subsequent implant loosening. Aseptic loosening is a major cause of the failure of megaprostheses [7], and the novel stem used here may overcome some of the limitations of traditional fixation methods. 

 The threaded stem design allows for simple, controlled insertion of the stem. Because it does not rely on a tight “scratch-fit” for initial stability like most press-fit stems, there is greater tolerance in the difference between the existing or prepared canal diameter and the stem diameter. This was an advantage in this case because the stem needed to be custom made and a particular diameter needed to be chosen even though the existing canal could not necessarily be exactly ascertained beforehand. If this novel stem is used in a typical fashion for a long bone megaprosthesis, insertion of the stem with a simple screw-in method that is highly tactile and familiar to surgeons compared to the forceful impaction of a standard press-fit stem will likely reduce the risk of intraoperative fracture. Once the stem is fully seated and the collar is compressed against the face of the osteotomy, interlocking pins or screws can be used to hold it in place and prevent derotation. The smooth threaded stem design not only provides immediate axial and rotational stability but also allows for easy extraction of the implant if it should become infected. The surface of the stem is designed to promote proximal bony ingrowth, prosthetic integration for long-term fixation, and preservation of bone stock. It is a solid stem with a relatively large diameter compared to the COMPRESS® and should be highly resistant to stem fracture.

With the use of computer-aided design and three-dimensional (3D) printing, custom-designed megaprostheses have re-emerged as a viable, albeit expensive, option for the reconstruction of massive bone defects after tumor resection. Although modular off-the-shelf implants have largely replaced the original custom-designed implants of the 1980s that took weeks to manufacture and necessitated neoadjuvant chemotherapy, custom implants have found a useful niche in the current limb salvage paradigm in parts of the world wealthy enough to afford them. By harnessing the creativity and experience of surgeons with the technology and tools of modern engineering and manufacturing, unique situations for which there are no standard reconstructive solutions can be solved on an individual basis. Large studies will likely be impossible due to the uniqueness of each situation, but case reports and small case series to promote knowledge sharing will facilitate further improvement upon prior designs and avoid needless redundancy. 

## Conclusions

Malignant transformation of enchondromas in Ollier’s disease can create challenging reconstructive situations after surgical resection. The novel stem design presented here was relatively straightforward to implant and demonstrated long-term success despite difficult anatomical circumstances. It may be a custom option for other similarly unique situations or, in the future, an off-the-shelf possibility for standard megaprosthetic reconstructions. 

## References

[1] Silve C, Jüppner H (2006). Ollier disease. Orphanet J Rare Dis.

[2] Verdegaal SH, Bovée JV, Pansuriya TC (2011). Incidence, predictive factors, and prognosis of chondrosarcoma in patients with Ollier disease and Maffucci syndrome: an international multicenter study of 161 patients. Oncologist.

[3] Schwartz HS, Zimmerman NB, Simon MA, Wroble RR, Millar EA, Bonfiglio M (1987). The malignant potential of enchondromatosis. J Bone Joint Surg Am.

[4] Healey JH, Morris CD, Athanasian EA, Boland PJ (2013). Compress knee arthroplasty has 80% 10-year survivorship and novel forms of bone failure. Clin Orthop Relat Res.

[5] Ng VY, Louie P, Punt S, Conrad EU (2017). Allograft reconstruction for sarcomas of the tibia. Open Orthop J.

[6] Mazaleyrat M, Le Nail LR, Auberger G (2020). Survival and complications in hinged knee reconstruction prostheses after distal femoral or proximal tibial tumor resection: a retrospective study of 161 cases. Orthop Traumatol Surg Res.

[7] Zhang HR (2022). Application and development of megaprostheses in limb salvage for bone tumors around the knee joint. Cancer Control.

